# ROS Plays a Role in the Neonatal Rat Intestinal Barrier Damages Induced by Hyperoxia

**DOI:** 10.1155/2020/8819195

**Published:** 2020-12-26

**Authors:** D. Y. Liu, W. J. Lou, D. Y. Zhang, S. Y. Sun

**Affiliations:** ^1^ShengJing Hospital of China Medical University Department of Gastroenterology and Medical Research Center, Liaoning Key Laboratory of Research and Application of Animal Models for Environmental and Metabolic Diseases, SanHao Street #36, HePing District, ShenYang 110000, China; ^2^ShengJing Hospital of China Medical University Department of Gastroenterology, SanHao Street #36, HePing District, ShenYang 110000, China

## Abstract

**Background:**

Hyperoxia treats a subset of critical neonatal illnesses but induces intestinal damage in neonatal pups. In this process, the intestinal flora and mucosal epithelium might be altered by hyperoxia. So the changes of the intestinal flora and mucosal epithelium were studied.

**Methods:**

Neonatal rats were randomized into the model group that was exposed to hyperoxia and the control group that was maintained under normoxic conditions; then, intestinal lavage fluid and intestinal tissues were harvested. ELISA was used to detect D-lactic acid (D-LA), endotoxin (ET), diamine oxidase (DAO), intestinal fatty acid binding protein (i-FABP), liver-type fatty acid binding protein (L-FABP) and cytokines in the intestinal lavage of neonatal rats during hyperoxia. The intestinal zonula occluden-1 (ZO-1), occlusion protein (Occludin), and closure protein-4 (Claudin-4) of neonatal pups were detected by immunohistochemistry, western blotting, and real-time Polymerase chain reaction (RT-PCR) during hyperoxia. NCM460 cell survival rates were assayed by 3-(4,5-dimethylthiazol-2-yl)-2,5-diphenyltetrazolium bromide (MTT) during hyperoxia and administration of N-acetyl-L-cysteine (NAC). The expression levels of ZO-1, Occludin, and Claudin-4 in NCM460 cells were detected by immunohistochemistry, western blotting, and RT-PCR during hyperoxia and NAC.

**Results:**

D-LA, ET, L-FABP, i-FABP, DAO, TNF-*α*, IL-10, and IFN-*γ* were significantly increased by hyperoxia, while ZO-1, Occludin, and Claudin-4 were clearly decreased in the hyperoxia group compared with the control group. NAC promoted cell survival, which was inhibited by hyperoxia. The cellular expression levels of ZO-1, Occludin, and Claudin-4, which were lowered by hyperoxia, were increased by NAC.

**Conclusion:**

Hyperoxia causes injury of the intestinal mucosa, and ROS plays a role in this intestinal damage during hyperoxia.

## 1. Background

Hyperoxia (continuously inhaling a high concentration of oxygen) is essential for the treatment of several critical neonatal illnesses. However, hyperoxia creates intestinal serosal and submucosal vasodilation, vascularization, and growth retardation in neonatal rats [1]. Because in neonatal rats the intestinal villi and mucosa continue to grow and differentiate after birth, the long-term effects of postnatal hyperoxia exposure may induce the intestinal barrier to change [2, 3].

The intestinal tight junction (TJ) is a type of protein complex that connects intestinal epithelial cells near the apical surface. TJ is composed of three membrane proteins including the occlusion protein (Occludin), the closure protein (Claudin), and the junction adhesion and stent protein zonula occluden-1 (ZO-1). These membrane proteins are connected to the cytoskeleton through stent proteins to maintain the integrity of the TJ. The TJ is a physical barrier that can prevent the absorption of harmful molecules such as pathogens, toxins, and allergens. TJ destruction is the pathological basis of many intestinal diseases.

Cytokines are regulatory peptides that can be produced by many cells. Cytokines are involved in cell differentiation, proliferation, activation, and interactions. Cytokines often possess overlapping biological activities and exert different effects at different concentrations [4]. And many cytokines play key roles during hyperoxia [4, 5].

We previously found that intestinal secretory IgA (SIgA) and IgA changed in neonatal rats during hyperoxia [3]. Thus, we hypothesized that the intestinal flora and mucosal epithelium might be altered by hyperoxia. To test this hypothesis, D-lactic acid (D-LA) and endotoxin (ET) were detected to determine changes in the intestinal bacteria of neonatal rats. In addition, diamine oxidase (DAO), intestinal fatty acid binding protein (i-FABP), and liver-type fatty acid binding protein (L-FABP), which are epithelial injury markers, along with ZO-1, Occludin, and Claudin-4 (intestinal TJs), were detected to determine changes in the intestinal mucosal barrier in neonatal rats. The intestinal cytokines interleukin-10 (IL-10), tumor necrosis factor-*α* (TNF-*α*), and interferon-*γ* (IFN-*γ*) can indicate hyperoxia-induced intestinal inflammation. Finally, intestinal epithelial cells were treated with antioxidants to determine if reactive oxygen species (ROS) were the cause of hyperoxia-induced intestinal damage in neonatal rats.

## 2. Materials and Methods

### 2.1. Animal Model

The animal model used was described previously [3, 6]. Briefly, timed pregnant Sprague-Dawley rats, which were obtained from the Animal Center of Shengjing Hospital of China Medical University (Shenyang, China), were individually housed in transparent cages. Then, the pups were randomly divided into the control group (exposed to atmospheric air, FiO_2_ = 0.21, *N* = 60) and the hyperoxia group (exposed to 90~95% O_2_, FiO_2_ = 0.9 ~ 0.95, *N* = 203) within 12 h of birth. In the hyperoxia group, oxygen was continuously delivered into sealed environmental chambers to achieve a constant concentration of 90~95% oxygen, as confirmed daily by an oxygen monitor (OM-25ME, Maxtec, Salt Lake City, UT, USA). Oxygen and atmospheric air were filtered through natrolite to maintain the CO_2_ concentration below 0.5% (as confirmed using a DapexGas Monitor, American). To equalize the effect of nursing on the pups' development and to eliminate maternal effects between groups (from the control group to the hyperoxia group and vice versa), the nursing mothers were switched every 24 h. The chambers were open for 1 h/d to switch the nursing mothers. Room temperature (25~27°C), humidity (70%~80%), and daily light-dark cycles were automatically controlled. All animal husbandry, handling, and procedures were reviewed and approved to conform to the animal Ethics Committee of China Medical University guidelines. At neonatal days 1, 3, 5, 7, 10, and 14, the pups were sacrificed by an intraperitoneal injection of pentobarbital sodium (50 mg/kg); intestinal lavage fluid and intestinal tissues were then harvested.

### 2.2. Intestinal Lavage

Lavage fluid was collected as described previously [6]. Briefly, the sacrificed neonatal rats were placed in a supine position. The small intestine was taken out; then, the distal end of the small intestine was ligated and 1 ml ice-cold normal saline was gently instilled into the small intestine at the pyloric end via a syringe. Normal saline was withdrawn and collected. Each animal was lavaged five times. The lavage fluid was then centrifuged to remove cells and debris. Supernatants were stored at -80°C.

### 2.3. Cell Culture

The human normal colon mucosal epithelial cell line NCM460 (C335) was obtained from the Shanghai Institute of Cell Biological,Chinese Academy of Sciences. The cells were incubated at 37°C in air and 5% CO_2_ in sterile basal medium/DMEM-H (Sigma-Aldrich, St. Louis, MO, USA) supplemented with 10% fetal bovine serum (Genview, Beijing, China), 1% L-glutamine, 100 U/ml penicillin, 100 mg/ml streptomycin, and 0.25 mg/ml amphotericin B. The culture medium was changed every 2 to 3 d. Prior to treatment, the cells (1 × 10^6^ cells/ml) were plated with fresh medium and cultured in 37°C in air and 5% CO_2_ for 24 h. On the second day after plating, the cells were treated with N-acetyl-L-cysteine (NAC) or apocynin (APO) and/or 85% oxygen (three-gas incubator, CB160, BINDER, Neckarsulm, Germany) for 24 h. The control cells received no treatment. Next, we harvested the cultured cells and extracted RNA and protein from the cells. All experiments were repeated 6 to 8 times.

### 2.4. Cell Survival Analysis by 3-(4,5-Dimethylthiazol-2-Yl)-2,5-Diphenyltetrazolium Bromide (MTT) Assay

Prior to treatment, the cells were plated in 96-well microtiter plates with fresh medium and cultured in 37°C in air and 5% CO_2_ for 24 h. On the second day after plating, the cells were treated with NAC (5, 10, or 20 *μ*M) or APO (5, 10, or 20 *μ*M) and/or 85% hyperoxia for 24 h. The cells treated with phosphate-buffered saline (PBS) or ethanol were used as the control group, respectively. Next, the cells were treated with 20 *μ*l MTT for 4 h at 37°C. The reactions were stopped by adding DMSO. We determined the absorbance of each well at 450 nm. Each sample was tested 6 to 8 times.

### 2.5. Enzyme-Linked Immunosorbent Assay (ELISA)

ELISA kits were used to measure the concentrations of D-LA, ET (also known as lipopolysaccharide, LPS), L-FABP, i-FABP, DAO, TNF-*α*, IFN-*γ*, and IL-10 (CUSABIO, Wuhan, China) in intestinal lavage fluid, according to the manufacturer's instructions. The absorbance of each solution was determined at a wavelength of 450 nm. Assay sensitivities were determined as follows: 7.8 ng/ml for D-LA, 0.78 pg/ml for ET, 1.56 pg/ml for L-FABP, 0.312 ng/ml for i-FABP, 0.195 mIU/ml for DAO, 1.56 pg/ml for TNF-*α*, 0.156 pg/ml for IFN-*γ*, and 0.78 pg/ml for IL-10. Intra-assay and interassay coefficients were 8% and 10%, respectively, for D-LA, ET, L-FABP, i-FABP, DAO, TNF-*α*, IFN-*γ*, and IL-10.

### 2.6. Immunohistochemical Staining of Intestinal ZO-1, Occludin, and Claudin-4

Paraffin-embedded intestinal sections were deparaffinized, rehydrated, and incubated with rabbit anti-rat ZO-1 (1 : 200, catalog No. 21773-1-AP, Proteintech, Rosemont, IL, USA), Occludin (1 : 200, catalog No. 13409-1-AP, Proteintech, Rosemont, IL, USA), and Claudin-4 (1 : 200, catalog No. 16195-1-AP, Proteintech, Rosemont, IL, USA) by sequential incubation. The slides were rinsed three times with pH 7.4 0.02 M PBS after each incubation, and the sections were counterstained with hematoxylin. The sections were observed using a digital camera (Olympus Corp., Tokyo, Japan) attached to a light microscope at a magnification of 200x. The primary antibody was replaced with PBS as a negative control. The absorbance values of ZO-1, Occludin, and Claudin-4 were compared using GraphPad Prism version 6.0 software (GraphPad Software, Inc., La Jolla, CA, USA) after scanning.

### 2.7. ZO-1, Occludin, and Claudin-4 Protein of Intestine Tissues and of NCM460 Cells Analyzed by Western Blotting

Proteins were extracted from intestinal tissues and NCM460 cells according to the manufacturer's instructions (Beyotime Institute of Biotechnology, Shanghai, China), and protein concentrations were determined with a BCA Protein Assay Kit according to the manufacturer's instructions (P0013C, Beyotime Institute of Biotechnology). The proteins (40 g) were separated by sodium dodecyl sulfate (SDS) polyacrylamide gel electrophoresis and transferred to polyvinylidene fluoride membranes. The membranes were blocked with Tris buffer containing 50 ng/l skim milk and probed with rabbit anti-ZO-1 (1 : 1000, catalog No. bs-1329R, Bioss, China), rabbit anti-Occludin (1 : 1000, catalog No. WL01996, Wanleibio, China), rabbit anti-Claudin-4 (1 : 1000, catalog No. bs-2790R, Bioss, China), or rabbit anti-*β*-actin (1 : 1000, catalog No. WL01372, Wanleibio, China) followed by a peroxidase-conjugated secondary antibody. Subsequently, the membranes were incubated with an enhanced chemiluminescent substrate (Thermo Fisher Scientific, Inc., Waltham, MA, USA), and the images were captured using a c300 Gel Imaging System (Azure Biosystems, Inc., Dublin, CA, USA). A Tanon 2500 Fully Automatic Digital Gel Imaging System (Tanon Science and Technology Co., Ltd., Shanghai, China) was used to scan images and to analyze the densitometry values of ZO-1, Occludin, and Claudin-4 protein levels as normalized to *β*-actin.

### 2.8. Real-Time Polymerase Chain Reaction (Real-Time PCR) Analysis of ZO-1, Occludin, and Claudin-4 mRNA in the Intestine Tissues and in NCM460 Cells

Total RNA was extracted from intestinal tissues and NCM460 cells using Trizol. cDNA was synthesized using 100 ng RNA (RR420A, TaKaRa Biotechnology Co., Dalian, China). RT-PCR was performed using an RNA Mini Kit (RR047A, TaKaRa Biotechnology Co., Dalian, China) in a LightCycler® 480 Real-Time PCR System (Roche, Basel, Switzerland). The primers for ZO-1, Occludin, and Claudin-4 are as follows:

1. Rat ZO-1-F: 5′-GGAAGCTATATGAACGGTCTCA-3′
2. Rat ZO-1-R: 5′-CGATCATCATGCAAATCAAGGT-3′
3. Rat Occludin-F: 5′-CCATCTGCAGCCAGTTTTATAC-3′
4. Rat Occludin-R: 5′-GTCCATCTTTCTTCGGGTTTTC-3′
5. Rat Claudin-4-F: 5′-GATGCTTCTCTCAGTCGTAGG-3′
6. Rat Claudin-4-R: 5′-GCACCATAATCAGCATGCTTG-3′
7. Rat *β*-actin-F: 5′-TGTCACCAACTGGGACGATA-3′
8. Rat *β*-actin-R: 5′-GGGGTGTTGAAGGTCTCAAA-3′
9. Human ZO-1-F: 5′-AAAGAGAAAGGTGAAACACTGC-3′
10. Human ZO-1-R: 5′-TTTTAGAGCAAAAGACCAACCG-3′
11. Human Occludin-F: 5′-AACTTCGCCTGTGGATGACTTCAG-3′
12. Human Occludin-R: 5′-GACTCGCCGCCAGTTGTGTAG-3′
13. Human Claudin-4-F: 5′-CCACAACATCATCCAAGACTTC-3′
14. Human Claudin-4-R: 5′-CAGAATACTTGGCGGAGTAAGG-3′
15. Human *β*-actin-F: 5′-CCTGGCACCCAGCACAAT-3′
16. Human *β*-actin-R: 5′-GGGCCGGACTCGTCATAC-3′

The primers and fluorescent probes for ZO-1, Occludin, Claudin-4, and the internal reference (*β*-actin) were purchased from TaKaRa (Dalian, China). The PCR conditions were as follows: a preliminary cycle at 95°C for 10 s followed by 45 cycles of 95°C for 5 s and 60°C for 20 s, then 1 min at 60°C and 5 s at 95°C. We confirmed that the efficiency of amplification for each target gene (*β*-actin) was 100% in the exponential phase of PCR. mRNA levels were normalized to *β*-actin levels according to the following formula: active levels of target (ZO-1, Occludin, and Claudin-4) mRNA = 2^−ΔΔCt^ × 100%. ΔΔCt = (hyperoxia CT_target_ − CT_*β*−actin_) − (control CT_target_ − CT_*β*−actin_). The mRNA levels of ZO-1, Occludin, and Claudin-4 in intestinal tissues or in NCM460 cells exposed to hyperoxia were compared with those of control intestinal tissues or cells, respectively.

### 2.9. Statistical Analysis

For each experiment, we tested at least ten generations of each group. The data from all groups are reported as the means ± standard deviations. All statistical analyses were performed using SPSS version 25.0 software (IBM Corp., Armonk, NY, USA). The statistical significance of the data was determined using the unpaired, two-tailed Student's *t*-test and one-way analysis of variance with the Dunnett test. Values of *P* < 0.05 were considered statistically significant.

## 3. Results

### 3.1. D-LA, ET, L-FABP, i-FABP, and DAO Contents in Neonatal Rat Intestinal Lavage

**Figure 1:**
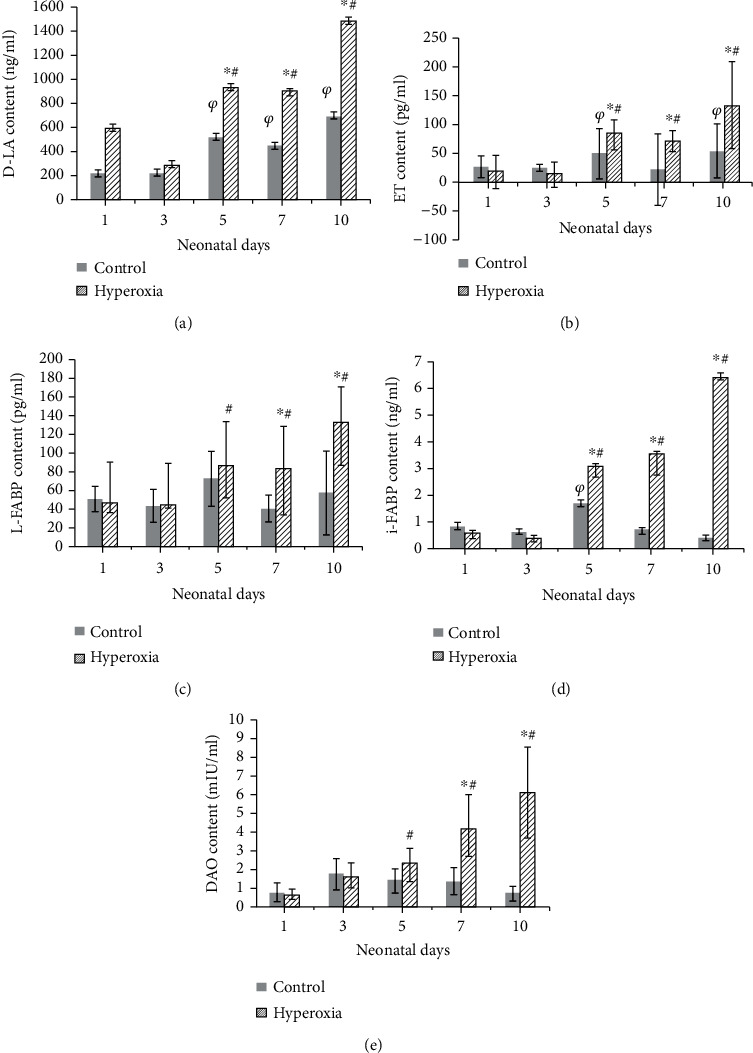
The levels of D-LA, ET, L-FABP, i-FABP, and DAO in intestinal lavage fluid. (a) D-LA; (b) ET; (c) L-FABP; (d) i-FABP; (e) DAO. As newborn rats grew and developed, D-LA, ET, L-FABP, i-FABP, and DAO levels gradually increased (^#^*P* < 0.05 or *P* < 0.01) in the hyperoxia group. But in the control group, only D-LA and ET gradually increased (^*φ*^*P* < 0.05 or *P* < 0.01). In the control group, i-FABP levels in 5-day-old neonatal pups increased compared to the other pups. Compared with the control group, D-LA, ET, L-FABP, i-FABP, and DAO levels increased significantly in the hyperoxia group (^∗^*P* < 0.05 or *P* < 0.01) (*n* = 10 at each time point).

As newborn rats grew and developed, in the hyperoxia group, D-LA (Figure 1(a)), ET (Figure 1(b)), L-FABP (Figure 1(c)), i-FABP (Figure 1(d)), and DAO (Figure 1(e)) gradually increased, reaching the highest levels at neonatal day 10. In the control group, however, only D-LA and ET levels gradually increased with the growth and development of newborn pups, and compared to the other pups, the i-FABP levels of 5-day-old neonatal pups increased but were obviously lower than those of the hyperoxia group. Overall, compared with the control group, D-LA, ET, L-FABP, i-FABP, and DAO levels increased significantly in the hyperoxia group (*P* < 0.05 or *P* < 0.01).

### 3.2. TNF-*α*, IFN-*γ*, and IL-10 Levels in Neonatal Rat Intestinal Lavage

**Figure 2:**
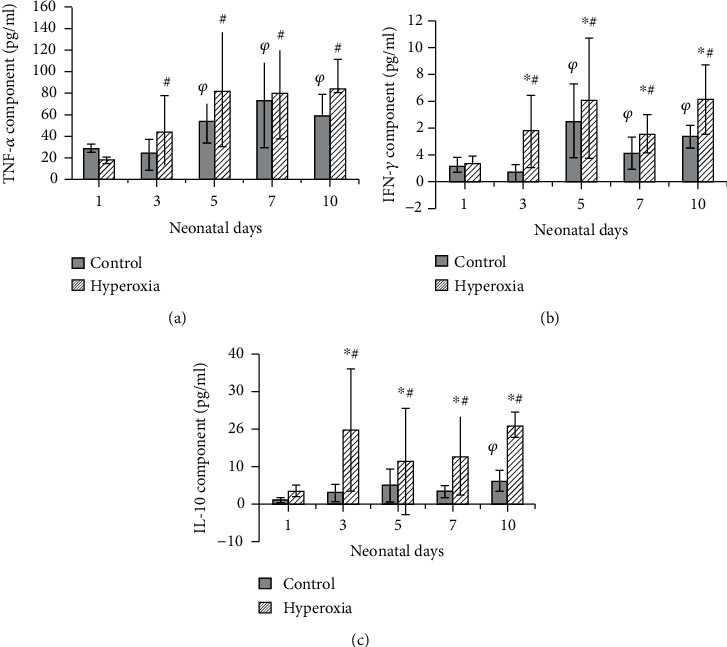
The levels of TNF-*α*, IFN-*γ*, and IL-10 in intestinal lavage fluid. (a) TNF-*α*; (b) IFN-*γ*; (c) IL-10. As newborn rats grew and developed, TNF-*α*, IFN-*γ*, and IL-10 levels in neonatal rat intestinal lavage gradually increased in both the control group (^*φ*^*P* < 0.05 or *P* < 0.01) and the hyperoxia group (^#^*P* < 0.05 or *P* < 0.01). Compared to the control group, IL-10 and IFN-*γ* levels were significantly elevated in the hyperoxia group (^∗^*P* < 0.05 or *P* < 0.01) (*n* = 10 in each time point).

As newborn rats grew and developed, TNF-*α* (Figure 2(a)), IFN-*γ* (Figure 2(b)), and IL-10 (Figure 2(c)) levels in neonatal rat intestinal lavage gradually increased. On neonatal days 5, 7, and 10, TNF-*α* and IFN-*γ* levels were clearly higher than on neonatal days 1 and 3, in both the control group and the hyperoxia group. In the control group, IL-10 clearly increased on neonatal day 10 only. And in the hyperoxia group, IL-10 was significantly elevated on neonatal days 3, 5, 7, and 10. Compared with the control group, IL-10 and IFN-*γ* significantly increased in the hyperoxia group (*P* < 0.05 or *P* < 0.01), but the increase in TNF-*α* did not achieve statistical significance.

### 3.3. Immunohistochemical Staining of Neonatal Rat Intestinal ZO-1, Occludin, and Claudin-4

**Figure 3:**
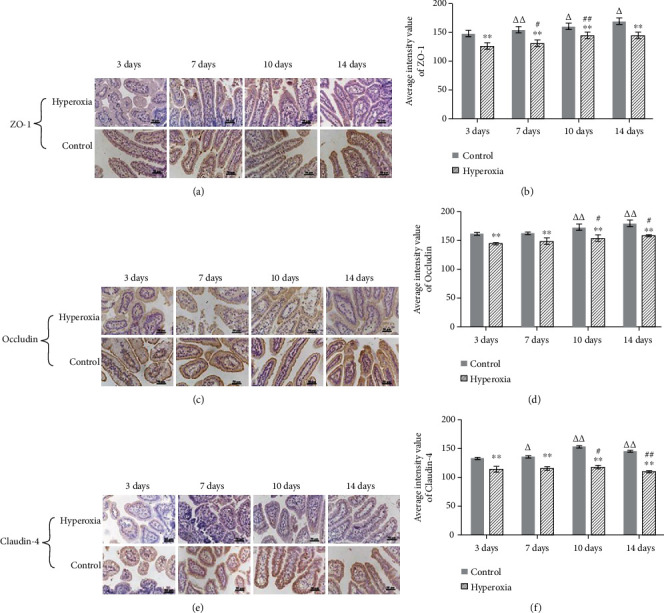
The expression of the intestinal tight junction proteins ZO-1, Occludin, and Claudin-4 in neonatal rats treated with hyperoxia. ZO-1, Occludin, and Claudin-4 located on the cell membrane and in the cytoplasm. Compared to the control group, ZO-1, Occludin, and Claudin-4 expressions were significantly decreased in the hyperoxia group (^∗∗^*P* < 0.01). As newborn rats grew and developed, ZO-1, Occludin, and Claudin-4 levels gradually increased (^Δ^*P* < 0.05 or ^ΔΔ^*P* < 0.01) in the control group. In the hyperoxia group, as newborn rats grew and developed, ZO-1 and Occludin levels gradually increased, but Claudin-4 was only elevated on neonatal day 10, and decreased on neonatal day 14 (^#^*P* < 0.05 or ^##^*P* < 0.01) (*n* = 10 at each time point).

We used immunohistochemical staining to investigate ZO-1, Occludin, and Claudin-4 localizations and expressions in the rat intestine. Results showed that ZO-1 (Figure 3(a)), Occludin (Figure 3(c)), and Claudin-4 (Figure 3(e)) were highly localized on the cell membrane and in the cytoplasm. As newborn rats grew and developed, ZO-1 (Figure 3(b)), Occludin (Figure 3(d)), and Claudin-4 (Figure 3(f)) levels gradually increased (*P* < 0.05 or *P* < 0.01) in the control group. In the hyperoxia group, ZO-1 and Occludin levels gradually increased as newborn rats grew and developed, but Claudin-4 was only elevated on neonatal day 10, then decreased on neonatal day 14 (*P* < 0.05 or *P* < 0.01). Compared with the control group, expression levels of ZO-1, Occludin, and Claudin-4 decreased on neonatal days 3, 7, 10, and 14 in the hyperoxia group (*P* < 0.05 or *P* < 0.01).

### 3.4. Hyperoxia Affects the Levels of Neonatal Rat Intestinal ZO-1, Occludin, and Claudin-4 Proteins

**Figure 4:**
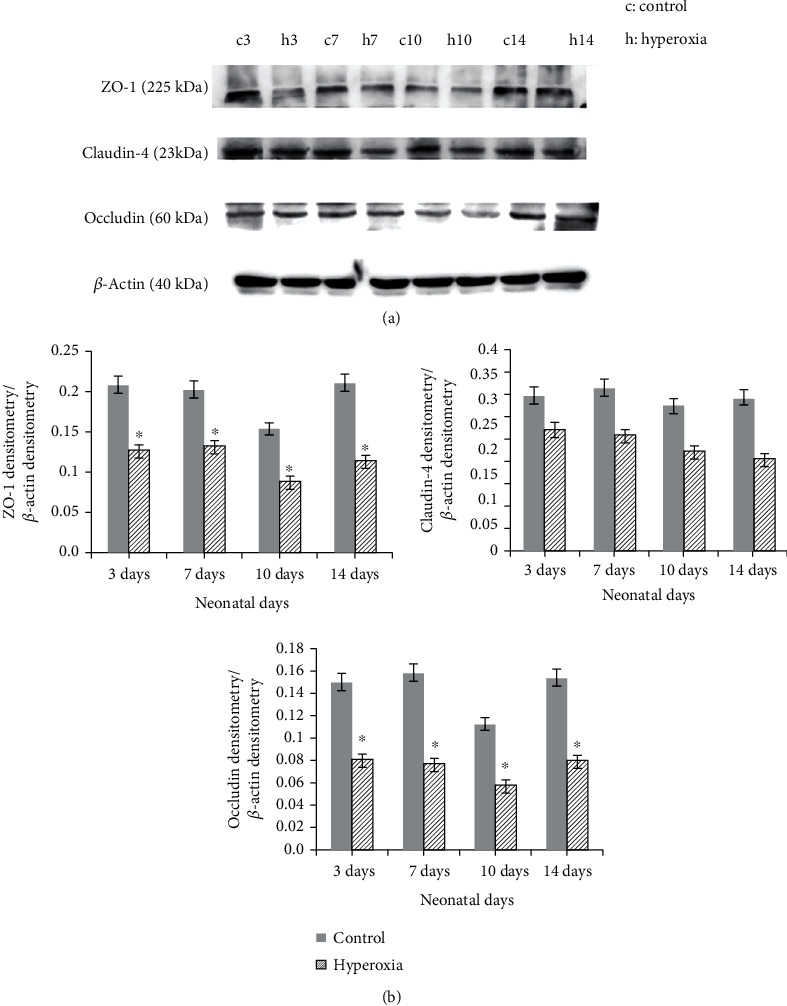
Hyperoxia affects the expression of intestinal ZO-1, Occludin, and Claudin-4 protein. (a) Specific bands for ZO-1, Occludin, and Claudin-4. (b) Densitometric analysis of Claudin-4, Occludin, and ZO-1 protein. Compared to the control group, the expressions of ZO-1, Occludin, and Claudin-4 were significantly decreased in the hyperoxia group (^∗^*P* < 0.05 or *P* < 0.01; *n* ≥ 10 in each group). *β*-Actin served as an internal control. Densitometric measurements were used to analyze ZO-1, Occludin, and Claudin-4 protein levels normalized to that of *β*-actin.

Protein expression levels of ZO-1, Occludin, and Claudin-4 were measured by western blotting. As indicated in Figure 4(a), there were clear and specific bands of intestinal ZO-1 (225 kDa), Occludin (60 kDa), and Claudin-4 (23 kDa). In the hyperoxia group, the levels of these proteins were lower compared with the control group. Densitometry analysis (of ZO-1, Occludin, and Claudin-4/*β*-actin) revealed that the band intensity of each protein was significantly lower in the hyperoxia group compared with the control group (*P* < 0.05 or *P* < 0.01; Figure 4(b)).

### 3.5. Neonatal Rat Intestinal ZO-1, Occludin, and Claudin-4 mRNA Expression Levels

**Figure 5:**
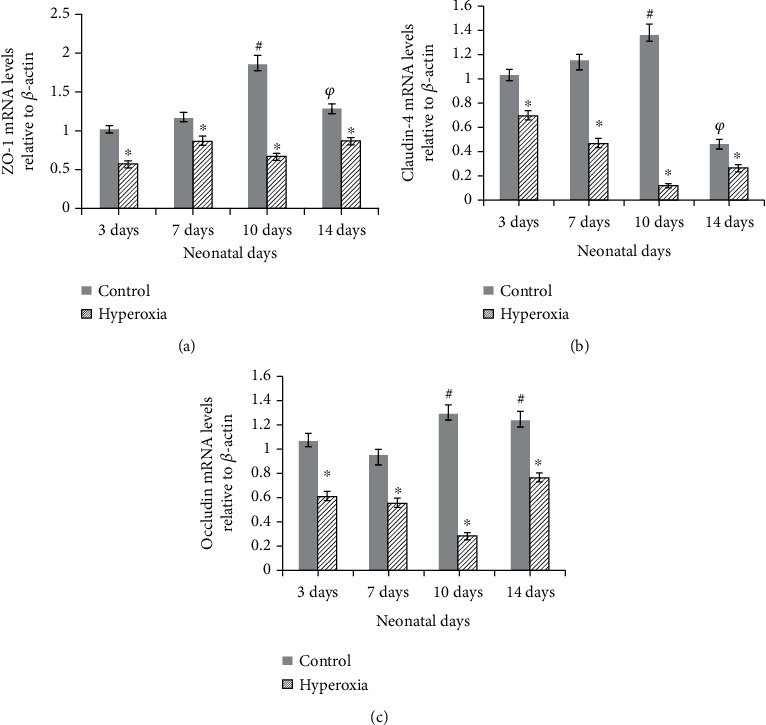
Hyperoxia affects the expression of intestinal ZO-1, Occludin, and Claudin-4 mRNA. Compared to the control group, ZO-1, Occludin, and Claudin-4 mRNA expression significantly decreased in the hyperoxia group (^∗^*P* < 0.05 or *P* < 0.01). In the control group, as newborn rats grew and developed, ZO-1, Occludin, and Claudin-4 mRNA expression levels gradually increased and reached peak levels on neonatal day 10 (^#^*P* < 0.05 or *P* < 0.01). There was then a downward trend on neonatal day 14 in ZO-1 and Claudin-4 mRNA levels (^*φ*^*P* < 0.05 or *P* < 0.01) (*n* ≥ 10 in each group). *β*-Actin served as an internal control.

As indicated in Figure 5, mRNA expression levels of intestinal ZO-1, Occludin, and Claudin-4 were similar to their respective protein expression levels in the hyperoxia group and the control group. In the hyperoxia group, intestinal ZO-1, Occludin, and Claudin-4 mRNA levels were significantly lower than those of the control group (*P* < 0.001). However, in the control group, as newborn rats grew and developed, ZO-1, Occludin, and Claudin-4 mRNA levels gradually increased, and reached peak levels on neonatal day 10 (*P* < 0.05 or *P* < 0.01). Then, on neonatal day 14, ZO-1 and Claudin-4 mRNA levels were reduced (*P* < 0.05 or *P* < 0.01).

### 3.6. The Effect of NAC and APO on Cell Survival

**Figure 6:**
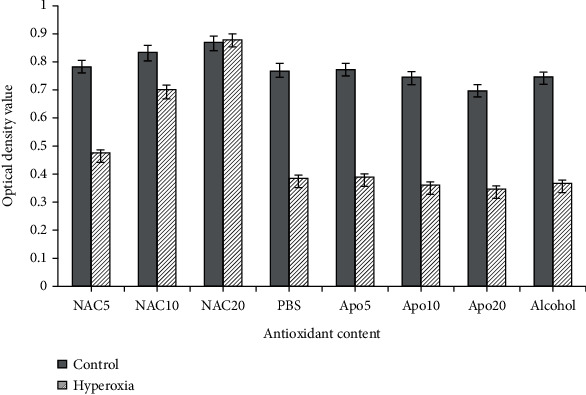
The effect of NAC and APO on cell survival. The survival rates of cells treated with PBS and ethanol were decreased significantly in the hyperoxia group as compared to the atmospheric air group. After the cells were treated with NAC, but not with APO, their survival rates increased in a dose-dependent manner (*n* ≥ 6 in each group).

In our previous studies, ROS was speculated to play a key role in hyperoxia [5, 7]. Therefore, in this study, the ROS inhibitor, NAC, which clears ROS from cells, and APO, which inhibits the production of cellular ROS, were used. As indicated in Figure 6, survival rates of cells treated with PBS or ethanol were significantly decreased during hyperoxia compared with control groups. After the cells in hyperoxia were treated with NAC, their survival rates gradually increased in a dose-dependent manner, while APO did not induce this effect. Therefore, NAC was used in subsequent experiments for treating cells.

### 3.7. The Protective Effect of NAC on NCM460 Cells under Hyperoxia

**Figure 7:**
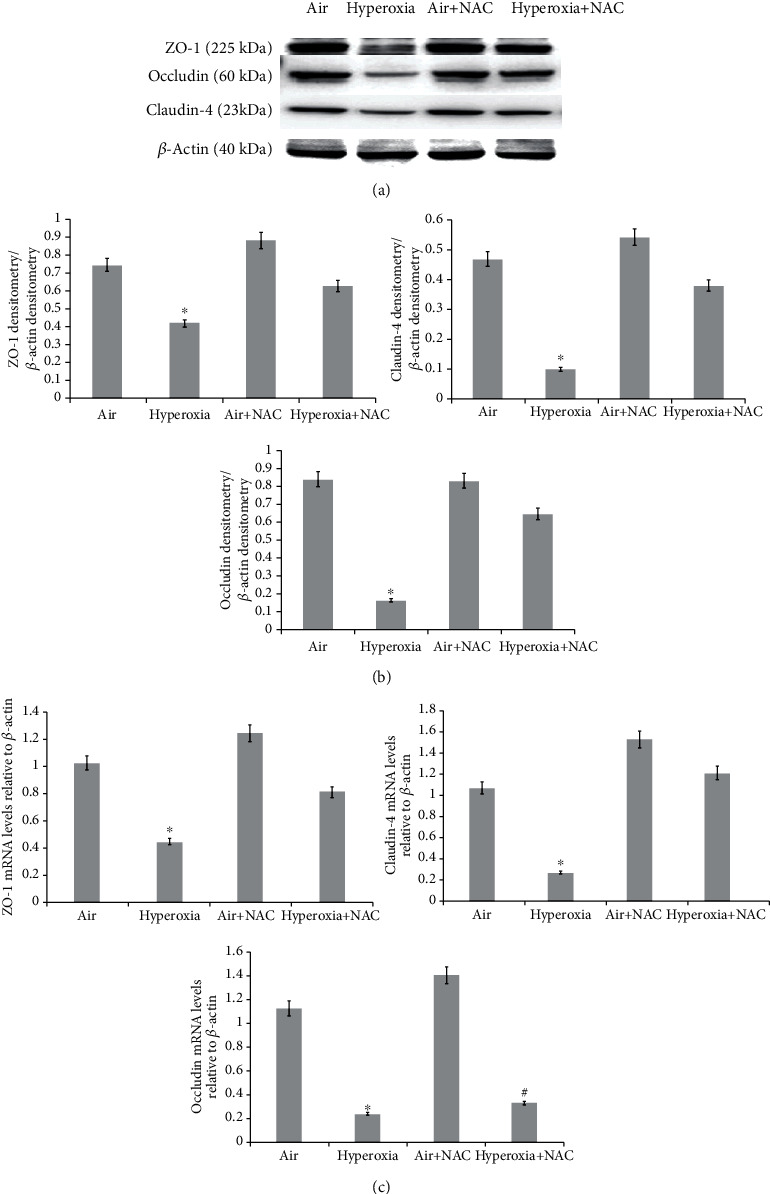
The protective effect of NAC on NCM460 cells treated with hyperoxia. (a) Specific bands for ZO-1, Occludin, and Claudin-4. (b) Densitometric analysis of Claudin-4, Occludin, and ZO-1. (c) Relative expression of ZO-1, Occludin, and Claudin-4 mRNA. Compared to the atmospheric air group, ZO-1, Occludin, and Claudin-4 expression levels significantly decreased in the hyperoxia group; however, after treatment with 20 mmol/l NAC, the levels of ZO-1, Occludin, and Claudin-4 gradually increased, and ZO-1 and Occludin came close to normal levels (^∗^*P* < 0.05 or *P* < 0.01 in the hyperoxia group compared to the atmospheric air group, the air + NAC group, and the hyperoxia + NAC group; ^#^*P* < 0.01 in the hyperoxia + NAC group compared to the atmospheric air group and the air + NAC group; *n* ≥ 6 in each group). *β*-Actin served as an internal control. Densitometric measurements were used to analyze ZO-1, Occludin, and Claudin-4 protein levels normalized to *β*-actin.

ZO-1, Occludin, and Claudin-4 protein (225 kDa, 60 kDa, and 23 kDa, respectively) from cells that were treated with/without NAC and/or 85% oxygen were detected (Figure 7(a)). Densitometry analysis (ZO-1, Occludin, and Claudin-4/*β*-actin) showed that expression levels of ZO-1, Occludin, and Claudin-4 were significantly reduced by hyperoxia, but they gradually came close to control levels after cells were treated with 20 mmol/l NAC (Figure 7(b)). ZO-1, Occludin, and Claudin-4 mRNA expression levels were similar to their respective protein levels in NCM460 cells (Figure 7(c)). In addition, mRNA expression levels of ZO-1, Occludin, and Claudin-4 were significantly decreased by hyperoxia, but returned to control levels after the cells were treated with 20 mmol/l NAC. Collectively, these results indicate that NAC plays a protective role in hyperoxia-induced intestinal injury. The protective role of NAC proves that ROS plays a key part in intestinal damage during hyperoxia.

## 4. Discussion

The intestinal barrier allows nutrient absorption and defends the body from penetration by dangerous macromolecules. Many factors can influence the intestinal barrier, such as gut microflora modifications, mucus layer alterations, and epithelial damage. Adequate function of the small intestine depends on the establishment and maintenance of compositionally distinct compartments that are lined by a monolayer of epithelial cells. The maintenance of a uniquely balanced microflora in the small intestine is essential to optimal health. The intestinal microbial flora includes a variety of microorganisms, predominantly bacteria, which colonize the gut of all living organisms. Shortly after birth, hundreds of species of bacteria establish themselves in the gastrointestinal (GI) tract. The GI tract is colonized by microorganisms from the oral cavity to the rectum.

This study analyzed several key indicators of intestinal health. D-LA mainly originates from bacterial production in the intestinal tract [8]. Thus, D-LA is used as a marker of bacterial infection. When intestinal bacteria are found at higher levels, more D-LA will be produced. The level of DAO activity is associated with the maturation and integrity of the small intestinal mucosa, and DAO activity is a useful biomarker for estimating the severity of intestinal mucosal disorders. L-FABP has a high affinity and capacity to bind to long-chain fatty acid oxidation products and may be an effective endogenous antioxidant. i-FABP is highly expressed in the intestinal epithelium and belongs to the family of soluble lipid binding proteins. L-FABP and i-FABP are thought to participate in most aspects of lipid biological activities by regulating the lipid availability for specific metabolic pathways, and by targeting and trafficking lipids to specific subcellular compartments [9]. L-FABP and i-FABP are useful markers for detecting the injury of the intestinal mucosa [10]. ET is a gram-negative bacterial cell wall component and a potent inducer of inflammatory cytokine production. In this study, D-LA and ET levels gradually increased as newborn rats grew and developed, and the i-FABP levels of 5-day-old neonatal pups increased also in the control group. This demonstrated that the intestinal flora of newborn rats was gradually established with the growth of newborn rats. However, it has been reported that D-LA, ET, TNF-*α*, and IL-6 levels, DAO activity, and bacterial translocation were increased in intestinal mucosa damage [11]. Our results showed that D-LA, ET, L-FABP, i-FABP, and DAO levels were significantly increased in the hyperoxia group compared to the control group. This indicated that hyperoxia caused injury of the intestine and disrupted the intestinal barrier in newborn rats [12].

Previous studies have shown an increase in inflammatory cells, proinflammatory factors, and immunological activation in hyperoxic conditions [6]. IFN-*γ* is secreted by Th1 cells and NK cells, and it activated M1 macrophages to secrete TNF-*α* [13]. IFN-*γ* is a proinflammatory factor that is primarily involved in the inflammatory response induced by hyperoxia [4, 14]. IL-10 is secreted by Th2 cells and M2 macrophages [13]. IL-10 is an anti-inflammatory cytokine that has antioxidant effects [13]. IL-10 may also reduce the mucosal inflammation that arises from Th1-secreted cytokines [15]. It has been reported that hyperoxia (100% oxygen) lowers the levels of TNF-*α* and increases the levels of IL-10 [16]. But another study showed that hyperoxia-exposed RV-infected mice showed increased expression of IFN-*γ* and TNF-*α* [17]. In our study, IFN-*γ* and IL-10 were markedly increased and TNF-*α* was increased only slightly under hyperoxia (95% oxygen), which implies that mucosal damage may be induced by the dysregulation of these cytokines. As an anti-inflammatory cytokine, IL-10 regulates the production of proinflammatory cytokines and inhibits TNF-*α* and other proinflammatory cytokines [18]. Therefore, we hypothesized that the increase in intestinal IL-10 might be a cause of low TNF-*α* levels. This proved that IL-10 can protect animals from damage due to hyperoxia-induced inflammation. However, the studies reported that under 65% hyperoxic conditions, IL-10 was significantly decreased in alveolar type II cells *in vitro* [19]. This may be because the neonatal intestine is not directly exposed in a high oxygen environment, but *in vitro*, these cells are directly exposed to hyperoxia, and therefore, the intestinal inflammatory response is delayed.

Intestinal epithelial integrity and barrier function are critical in the gut. To maintain the epithelial barrier, Occludin, Claudin-4, and ZO-1 form adherens and TJs between epithelial cells. In the gut, adherens and TJs between epithelial cells develop a continuous intercellular barrier that acts as the first physical barrier against a variety of pathogens and toxins to maintain intestinal homeostasis. ZO-1, which is expressed in epithelial cells, plays a key role in the maintenance and regulation of intestinal TJs. During intestinal epithelial barrier dysfunction, intestinal epithelial ZO-1 will relocate and exhibit expression changes. Occludin, which is expressed in epithelial cells, is also a TJ protein that is incorporated directly into TJ strands. Occludin not only is an essential component of TJs but also regulates paracellular pore and leak pathways through its effects on claudins [20]. It is widely accepted that Occludin could be a regulator of TJs [20]. Claudin proteins are fundamental components of TJs and comprise at least 24 proteins. Claudin-4, which is a claudin family member, may regulate pore pathways [20]. Both ZO-1 and Claudin-4 are reported to be involved in intestinal barrier dysfunction [21]. In this process, proinflammatory cytokines and ET play key roles; for example, TNF-*α* and ET can suppress Claudin-4 expression [22, 23]. Our previous results showed that, during hyperoxia, TNF-*α* and ROS were significantly elevated, and NF-*κ*B signaling was activated in intestinal epithelial cells [7]. As mentioned above, TNF-*α* and ET also increased in the intestinal mucosae of neonatal rats exposed to hyperoxia. These changes could affect the expression levels of ZO-1, Occludin, and Claudin-4. In this study, ZO-1, Occludin, and Claudin-4, which regulate the intestinal leaky pathway, were markedly decreased during hyperoxia *in vitro* and *in vivo*, which further supported the presence of gut barrier dysfunction in hyperoxia. This finding was in accordance with previous studies [12, 24]. This homogeneity showed that hyperoxia induced intestinal damage. Therefore, we think that, during hyperoxia, large amounts of TNF-*α* and ET are generated, which may, in turn, suppress Claudin-4 expression and thereby destroy TJs. It was previously reported that the intestinal TJ proteins, namely, ZO-1, Occludin, and Claudin-4, were significantly downregulated in necrotizing enterocolitis (NEC) [25]. Taken together, these findings suggest that the neonatal intestine exposed to hyperoxia could exhibit NEC.

As intestinal ROS were significantly elevated under hyperoxia [7], we next sought to verify the above results and to determine whether ROS are involved in the response to hyperoxia. It is common knowledge that there are two main sources of ROS generation: mitochondrial and cellular nicotinamide adenosine dinucleotide hydrophosphoric acid (NADPH). APO and NAC are two antioxidants that block ROS. APO blocks ROS production by NADPH oxidase. Conversely, NAC is an antioxidant that is generated from thiol-containing cysteine amino acid, and it is the precursor for glutathione, which scavenges oxygen free radicals in the body [26]. In other words, NAC is a scavenger of ROS [27], and NAC can clear ROS in various ways.

In order to verify the role of ROS in hyperoxia, NAC and APO were used to treat hyperoxic intestinal epithelial cells *in vitro*. MTT assay results showed that APO did not improve intestinal epithelial cell survival, but NAC could significantly increase NCM460 cell survival under hyperoxia. This could be because NAC can indirectly produce glutathione, which has antioxidant properties and can be directly used as an antioxidant to remove ROS [28]. APO only inhibits NADPH oxidase and does not scavenge ROS that are generated by hyperoxia. Therefore, NAC showed antioxidant activity, but APO did not. It was previously reported that NAC plays a protective role against oxidative stress [29]. Therefore, we selected NAC as an antioxidant to observe changes in ZO-1, Occludin, and Claudin-4 levels in NCM460 cells. The results showed that NAC treatment produced antioxidant activity in cells; thus, hyperoxia-induced suppression of ZO-1, Occludin, and Claudin-4 expression levels could be ameliorated by NAC. This demonstrated that the antioxidant NAC could play a protective role in intestinal epithelial cells during hyperoxia. This also confirmed that ROS participated in hyperoxia-induced intestinal damage.

## 5. Conclusions

ROS plays a role in the intestinal damage caused by hyperoxia, which affects the normal physiological structure and function of the intestine. Therefore, we can conclude that ROS contributes to the damage to the intestinal mucosa that occurs during hyperoxia.

## Data Availability

The data used to support the findings of this study are included within the article.

## Abbreviations

D-LA: D-Lactic acid
ET: Endotoxin
LPS: Lipopolysaccharide
DAO: Diamine oxidase
i-FABP: Intestinal fatty acid binding protein
L-FABP: Liver-type fatty acid binding protein
TJ: Tight junction
ZO-1: Zonula occluden-1
SIgA: Secretory IgA
IL-10: Interleukin-10
TNF-*α*: Tumor necrosis factor-*α*
IFN-*γ*: Interferon-*γ*
ROS: Reactive oxygen species
NAC: N-Acetyl-L-cysteine
APO: Apocynin
PBS: Phosphate-buffered saline
MTT: 3-(4,5-Dimethylthiazol-2-yl)-2, 5-diphenyltetrazolium bromide
ELISA: Enzyme-linked immunosorbent assay
Real-time PCR: Real-time polymerase chain reaction
GI: Gastrointestinal
Th: T-helper
NEC: Necrotizing enterocolitis
NADPH: Nicotinamide adenosine dinucleotide hydrophosphoric acid.
